# The effect of changing movement and posture using motion-sensor biofeedback, versus guidelines-based care, on the clinical outcomes of people with sub-acute or chronic low back pain*-a multicentre, cluster-randomised, placebo-controlled, pilot trial*

**DOI:** 10.1186/s12891-015-0591-5

**Published:** 2015-05-29

**Authors:** Peter Kent, Robert Laird, Terry Haines

**Affiliations:** 1 Institute of Sports Science and Clinical Biomechanics, University of Southern Denmark, Campusvej 55, Odense, M, 5230 Denmark; 2 Research Department, Spine Centre of Southern Denmark, Hospital Lillebaelt, Institute of Regional Health Services Research, University of Southern Denmark, Middelfart, Denmark; 3 Department of Physiotherapy, Monash University, Frankston, Victoria Australia; 4 Allied Health Research Unit, Monash Health, Clayton, Victoria Australia

**Keywords:** Low back pain, Rehabilitation, Movement, Posture, Clinical trial, Technology

## Abstract

**Background:**

The aims of this pilot trial were to (i) test the hypothesis that modifying patterns of painful lumbo-pelvic movement using motion-sensor biofeedback in people with low back pain would lead to reduced pain and activity limitation compared with guidelines-based care, and (ii) facilitate sample size calculations for a fully powered trial.

**Methods:**

A multicentre (8 clinics), cluster-randomised, placebo-controlled pilot trial compared two groups of patients seeking medical or physiotherapy primary care for sub-acute and chronic back pain. It was powered for longitudinal analysis, but not for adjusted single-time point comparisons. The intervention group (n = 58) received modification of movement patterns augmented by motion-sensor movement biofeedback (ViMove, dorsaVi.com) plus guidelines-based medical or physiotherapy care. The control group (n = 54) received a placebo (wearing the motion-sensors without biofeedback) plus guidelines-based medical or physiotherapy care.

Primary outcomes were self-reported pain intensity (VAS) and activity limitation (Roland Morris Disability Questionnaire (RMDQ), Patient Specific Functional Scale (PSFS)), all on 0–100 scales. Both groups received 6–8 treatment sessions. Outcomes were measured seven times during 10-weeks of treatment and at 12, 26 and 52 week follow-up, with 17.0 % dropout. Patients were not informed of group allocation or the study hypothesis.

**Results:**

Across one-year, there were significant between-group differences favouring the intervention group [generalized linear model coefficient (95 % CI): group effect RMDQ −7.1 (95 % CI–12.6;–1.6), PSFS −10.3 (−16.6; −3.9), QVAS −7.7 (−13.0; −2.4); and group by time effect differences (per 100 days) RMDQ −3.5 (−5.2; −2.2), PSFS −4.7 (−7.0; −2.5), QVAS −4.8 (−6.1; −3.5)], all p < 0.001. Risk ratios between groups of probability of improving by >30 % at 12-months = RMDQ 2.4 (95 % CI 1.5; 4.1), PSFS 2.5 (1.5; 4.0), QVAS 3.3 (1.8; 5.9).

The only device-related side-effects involved transient skin irritation from tape used to mount motion sensors.

**Conclusions:**

Individualised movement retraining using motion-sensor biofeedback resulted in significant and sustained improvements in pain and activity limitation that persisted after treatment finished. This pilot trial also refined the procedures and sample size requirements for a fully powered RCT.

This trial (Australian New Zealand Clinical Trials Registry NCT01572779) was equally funded by dorsaVi P/L and the Victorian State Government.

## Background

Low back pain (LBP) is highly prevalent and globally is the leading cause of disability, ahead of ischaemic heart disease, chronic obstructive pulmonary disease, major depressive illness, and other musculoskeletal disorders, including osteoarthritis [1]. It is also costly, both at a personal and societal level, with estimates of direct and indirect costs ranging from 0.4 % to 1.7 % of GDP, depending on the country and the econometric model used [2, 3].

Approximately 1 % of LBP in primary care is caused by serious pathology (cancer, spinal osteomyelitis, fracture, spinal stenosis, cauda equine syndrome, ankylosing spondylitis, visceral-referred pain) and approximately 20 % is due to nerve root irritation caused by disc disease or other forms of stenosis [4–6]. However, the majority of LBP seen in primary care is labelled ‘non-specific’ LBP, due to uncertainty about the accuracy and validity of other patho-anatomical diagnoses or descriptive labels, such as ‘facet syndrome’, ‘contained disc lesion’ or ‘instability’ [7].

Compared with placebo or no treatment, most non-surgical treatments for non-specific LBP show small to moderate effects, with one treatment showing little superiority over another [8]. In addition, short-term treatment effects typically reduce over the subsequent 12 months [9–11].

One explanation for this lack of demonstrated effect is that non-specific LBP is not one condition and that the wide heterogeneity of treatment response reflects clinically important subgroups with different treatment needs [12]. Therefore, mean differences in trials may conceal important effects in subgroups of patients [13]. This has resulted in considerable clinical and research interest in identifying such subgroups and better targeting of care for individual patients [14–17].

One of the approaches to individualised care is to target pain-related, dysfunctional movement patterns (muscle activation, lumbo-pelvic kinematic or postural patterns). Movement pattern aberrations reported in people with persistent LBP include increased trunk stiffness [18, 19], poor proprioception [20–22], postural dysfunction [23–25], and altered patterns of abdominal [26, 27] and extensor muscle activation [28–30]. Advice to stay active and exercise therapy are common key recommendations in LBP treatment guidelines [31–33] and their positive effects may be due to adaptive movement countering the potential for dysfunctional patterns to become habituated [34]. In addition, excessive loading is repeatedly implicated as a risk factor for back pain and this may occur for a variety of reasons, including protective movement patterns adopted during functional activity. For example, spending >5 % of the working day in >60 % of lumbar spine flexion is a risk factor for incident LBP (risk ratio 1.5) [35]. As a result, many intervention approaches are designed to target movement pattern aberrations associated with episodic and persistent LBP [36, 26, 37, 38, 16].

Translating kinematic and biomechanical findings from the laboratory to routine clinical practice is challenging and more complicated when the targeted movement patterns are diverse and subtle. It likely that such interventions would be facilitated by the use of technology but there have been limitations in the available non-invasive technology for measuring and monitoring movement patterns of individual patients in the clinic, especially during their normal activities of daily living. Similarly, there have been limitations to clinicians’ ability to provide accurate real-time feedback to people with LBP on the way they move during daily activities of work, rest and play. These limitations were constraining because there is evidence that such biofeedback can help people develop greater awareness of their activity and increase their voluntary control over otherwise involuntary processes [39].

Recently, new technology has resulted in wearable wireless motion-sensors that can quantify and analyse kinematic musculoskeletal function. This technology can assist in the evaluation of lumbopelvic movement patterns and postures, both in the clinic and in the patient’s daily functional activity (dorsaVi Ltd, Melbourne, Australia). These devices can also be easily programmed to provide individualised biofeedback to people with back pain to reinforce clinician-determined rehabilitation strategies in their everyday vocational, social and recreational activities, where changes to habituated movement behaviours most need to be reinforced. No previous clinical trials have investigated the effect of such technology-assisted approaches to the rehabilitation of lumbopelvic movement patterns.

Randomised controlled trials are the gold standard method for studying the effects of treatment and one type of trial design, cluster-randomised controlled trials, has some advantages in certain situations. Cluster-randomised trials, where randomisation occurs at the level of clinicians, practices, hospitals or geographic locations, instead of at the level of participating patients, have the advantage of better controlling for ‘contamination’ across clinicians or patients, where changing the behaviour or treatment of one person being studied may affect the behaviour or treatment of another [40]. Another advantage is that cluster trials typically focus on effectiveness, studying interventions in settings that more closely approximate their use in routine care. However, compared with individually randomised controlled trials, cluster trials need more participants to have the same statistical power and require more complex designs and methods of analysis [41, 42]. During the planning phase of a cluster trial, one of the requirements for determining the required sample size is to have an estimate of the statistical interdependence between individuals in the same cluster (intracluster/interclass correlation). Usually the best way to estimate this Intraclass Coefficient Correlation is to conduct a pilot study.

Therefore, the hypothesis investigated in this study was that ‘changing patterns of lumbo-pelvic movement and/or posture using motion-sensor biofeedback in people with LBP would lead to reduced pain and activity limitation, when compared with Guidelines-based medical or physiotherapy care and placebo. The aims of this cluster-randomised pilot clinical trial were to: (i) estimate the effect size and its variability, (ii) test the study protocol and procedures, and (iii) provide data to calculate sample size requirements that would allow adjusted individual time-point comparisons in a fully powered cluster-randomised clinical trial.

## Methods

### Trial design

This study was a multicentre, cluster-randomised, placebo-controlled, pilot clinical trial, with one-to-one allocation to intervention (Movement Biofeedback) and control (Guidelines-based Care) groups. The key elements of the protocol, including the primary outcome measures, were registered in the Australian New Zealand Clinical Trials Registry (NCT01572779) prior to the study commencing. The full trial protocol for this proof-of-concept study has not been published but is available on request from the first author (PK). As one function of a pilot study is to determine what to do when unforeseeable situations occur, we anticipated that protocol amendments would be required. Therefore, our strategy for managing them was to document every amendment, seek the approval of the relevant ethics committees for these changes, have independent external researchers provide project oversight, and have an independent external party audit the whole trial after it was completed, including adherence to the protocol.

### Participants

#### Inclusion and exclusion criteria

Patients were recruited by their treating clinicians. The inclusion criteria were any adult person aged between 18 and 65 years, presenting with a primary complaint of LBP (or back-related leg pain) with an average pain intensity of 3 or more on a 0–10 scale, and a LBP episode duration that was either sub-acute (3–12 weeks) or chronic (>12 weeks). Exclusion criteria were low back surgery or other invasive procedure within the previous 12 months, current pregnancy, severe hearing impairment, implanted electrical medical device, known allergic skin reaction to tapes and plasters, neoplasm, infection, inflammatory or neurological disorder, fracture or other joint or medically-related disorders.

Potential participants were informed about the study and given the option of providing written informed consent at the index consultation, or taking time to consider the decision and telling their clinician at the next consultation. All participants were advised that the purpose of the trial was to test if wearing the device would assist in the management of back pain but were not informed of the directional hypothesis being investigated.

#### Settings and locations where the data were collected and treated

The clinical sites where the trial was conducted were eight hospitals or outpatient primary care clinics in the State of Victoria in Australia. The Movement Biofeedback Group sites were: Austin Hospital – Heidelberg, Bounce Health Group – Ringwood, Olympic Park Sports Medicine Centre – Melbourne, The Clinic Werribee – Werribee. The Guidelines-based Care Group sites were: Epworth Hospital Richmond – Richmond, Stanlake Specialist Centre – Footscray, Myers Street Family Medical – Geelong, Peak Musculoskeletal – Hampton. The participating clinicians were two physicians, four GPs and three physiotherapists, all with a special interest in musculoskeletal conditions. The medical practitioners had an average of 25.8 years (SD6.9) post-graduate experience and the physiotherapists 19.0 years (SD 7.9). Clinics and clinicians were recruited by staff administering the trial.

### Randomisation

#### Randomisation level

In this cluster trial, randomisation only occurred at the level of clinics (clusters). As a result, clinicians at each clinic delivered only one type of treatment. Patient recruitment occurred from each clinician’s usual patient flow and clinicians were not blind to treatment.

#### Sequence generation

The random allocation of clusters (clinics) occurred in the following manner. Each of the three physiotherapy clinics was randomly paired with one of the medical clinics to form three pairs, and the remaining two medical clinics formed a fourth and final pair. Each pair was arbitrarily given a number from 1 to 4, and each pair contained an arbitrary Clinic A and Clinic B. These four numbered and paired codes, without clinic identification (blinded), were given to a researcher (TH) who generated a random number between 0.0 and 1.0 for Clinic A in each of the four pairs using Excel (Microsoft Corp, Redmond WA, USA). If the number was >0.5, Clinic A was assigned to be a Movement Biofeedback Group clinic and its paired Clinic B to be a Guidelines-based Care Group clinic. If the number was <0.5, the assignment direction was the reverse. This procedure resulted in one physician, one GP and two physiotherapists being randomised to the intervention (movement biofeedback) group and one physician, two GPs and one physiotherapist being randomised to the control (guidelines-based care) group.

#### Ethics

Ethics approval was obtained from three ethics committees: the Royal Australian College of General Practice (approval number NREEC 08/005, 11 February, 2009), Austin Health (H2009/03544, 25 August, 2011), and Epworth HealthCare (53111, 23 September, 2011). All recruited patients gave written informed consent.

#### Funding

Funding for this study was equally provided by (i) a grant from the Department of Business and Innovation (Market Validation Program), Victorian Government, Australia, and (ii) dorsaVi P/L (the Australian company who manufactures the ViMove motion-sensor system used in this study). The Department of Business and Innovation helped in the governance of the trial. DorsaVi supplied the motion-sensor equipment and coordinated the trial, assisted by a contract research organisation (Kendle P/L, Oakleigh, Victoria, Australia). All data and trial-related documentation were independently audited by Paul L Clark and Associates (Beaumaris Victoria, Australia). The authors analysed the results and wrote this paper independently of both funders, and neither funder had any influence over how these data were presented and the conclusions reached.

### Interventions

#### Both groups

All participants in both groups were assessed at baseline and attended a total of 6 (sub-acute episode duration patients) to 8 (chronic episode duration patients) consultations over a 10-week treatment period. They also received advice on staying active and general self-management of back pain. This advice was based on the 2003 Australian National Health and Medical Research Council guidelines for the management of Acute LBP [43], and European guidelines for the management of chronic non-specific LBP [44] in the absence of similar Australian guidelines for chronic LBP. The participants could also have received whatever usual medical and physiotherapy care was deemed essential by their clinicians, and such guidelines-based [44, 43] co-interventions were noted.

**Fig. 1:**
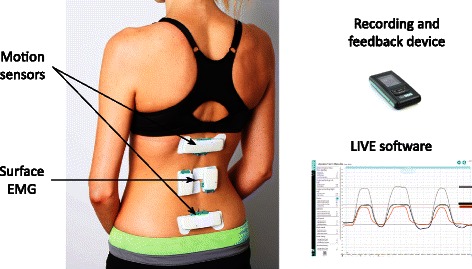
ViMove wearable motion-sensor system (this image has no copyright restrictions)

All participants wore the ViMove motion-sensor system (dorsaVi.com) for 4 to 10 hours in their activities of daily living, during and after each treatment session (6 to 8 times) over the 10-week treatment period. This system consists of: (i) two wireless motion-sensors that measure three-dimensional movement, movement velocity and acceleration, and orientation to gravity, (ii) two wireless surface electromyography (EMG) sensors that measure paraspinal muscle activation, (iii) a wireless recording device (approximately the size of a cigarette packet) that captures the sensor data, has a button that patients can push when an event occurs (such as an onset or increase in pain), an audio and vibration function that can be programmed to provide patient-specific biofeedback alerts, and (iv) a charging dock for these wireless devices. The system also has a comprehensive computer software application that clinicians use to observe movement characteristics in real-time, to download movement data from the recording device captured during activities of daily living, to analyse these data with the use of graphics-rich reports, and to compare an individual’s movement pattern with their previous assessments or with reference values. Using gyroscopes built into the two motion-sensors, the system also records whether the patient is sitting, standing, walking or lying down, at every time point during measurement. One motion-sensor is mounted on the thoraco-lumbar junction using a hypoallergenic, disposable adhesive pad and the other motion-sensor is mounted on the upper sacrum. This positioning allows isolation of the lumbar spine and pelvic components of three-dimensional lumbo-pelvic movement. The ViMove system has displayed good inter-tester (ICC (2,1) > 0.86) and intra-tester reliability (ICC(2,1) > 0.89) for lumbar movements [45] and excellent accuracy/concurrent validity with standard errors of measurement of 0.9° (95 % CI = ±1.8°) for the sagittal and 1.8° (3.6°) coronal planes [46] relative to the reference standard of the Optotrak 3D-motion tracking system (NaturalPoint Inc. Corvallis, Oregon USA) Fig. 1.

#### Movement Biofeedback Group

Patients in the Movement Biofeedback Group had an individualised assessment to determine whether, in their clinician’s judgement, there was a relationship between their movement or posture and their pain. This judgement was determined from the case history, the physical examination and the detailed kinematic information supplied by the ViMove system, worn both in the clinic and during the patient’s activities of daily living. Typically, this judgement involved the clinician identifying a dysfunctional movement pattern, changing the patient’s movement behaviour or posture and assessing whether there had been a change in pain.

The movement dysfunctions identified were diverse, but broadly could be classified into three potentially overlapping categories. Firstly, excessive end-range postural positions or repeated end-range movements, which may have occurred in sitting, standing, walking, or bending in any anatomical plane or combination of planes. For example, a person who performed sustained end range flexion by habitually sitting fully slumped, whose pain was relieved by assuming a more neutral sitting posture. Secondly, reduced muscle activation resulting in reduced load or stiffness control. For example, a person with inadequate hip and trunk muscle contribution during repetitive occupational bend and lifting. Thirdly, over-active muscle activation resulting in excessive load or stiffness control. For example a person whose lumbar flexion was restricted by excessive activity or guarding from superficial thoracolumbar extensor muscles, due to habituated fear of lumbar movement. These approaches to movement classification have been previously described in diverse subgrouping systems [47, 37, 48, 49, 38, 16].

The clinician then devised a patient-specific rehabilitation strategy designed to address any identified deficits in the patient’s pattern of lumbo-pelvic movement and/or posture. That strategy included up to three modes of intervention. Firstly, ‘live training’ in the clinic, where patients were instructed in how to alter their movement pattern (s) or posture using real-time on-screen biofeedback, while wearing the ViMove device. For example, using simple graphical feedback on a computer screen, patients could see in real-time their movement in the sagittal and frontal planes, the relative contributions of their lumbar spine and pelvic movement, and their habituated postural starting position. They could also be trained to perform movement in ways determined by the clinician to be more optimal. In this way, patients could gain a greater understanding of their own spinal kinematics and rehearse rehabilitation exercises that the clinician believed to be useful.

Secondly, using the ViMove software, clinicians could easily program motion-sensor biofeedback alerts (audio ‘beeps’ and/or vibration of the wireless recording device) that would occur during the 4- to 10- hours periods of the activities of daily living in which they wore the device. This biofeedback would prompt the patient when they ‘broke a rule’ that the clinician had programmed. For example, in the case of a patient who demonstrated painful slumped sitting posture, an alert would sound when seated lumbo-pelvic flexion exceeded a pre-determined angle for a sustained pre-determined period of time. Alternatively, and at clinician discretion, these alerts could have been triggered for a variety of other reasons, such as prompts to: (i) move following a prolonged period of postural inactivity, (ii) reduce the amount of end-range repeated or static loading, or (iii) perform recommended rehabilitation exercises.

Thirdly, specific exercises that supplemented the patient-specific movement biofeedback. For example, a patient whose habituated posture involved painful excessive lumbar spine extension (near end-range extension in a hyper-lordotic standing posture) would have been taught posterior pelvic tilt exercises and been encouraged to practise a less lordotic standing posture.

The intervention and the real-time movement biofeedback were recalibrated at each treatment session in response to the patient’s pain, clinical presentation and the movement information provided by the ViMove device. Therefore, the specific characteristics of the movement/posture targeted in the rehabilitation would change over time in response to the patient’s progress.

#### Guidelines-based Care Group

In addition to guidelines-based medical or physiotherapy care, the only other procedure undertaken by patients in the Guidelines-based Care Group was the wearing of the ViMove device 6 to 8 times over the 10-week treatment period. Their clinicians were blind to any motion-sensor/EMG information as the software reports were blocked via a software lock during the trial, with no capacity to program biofeedback for their patients. However, the ViMove system automatically uploaded the movement data to a central server so that it could be used by the researchers to compare to the movement characteristics of the Movement Biofeedback Group. Patients in the Guidelines-based Care Group were informed that the ViMove system was a measurement device.

The Movement Biofeedback Group and Guidelines-based Care Group treatments were similar to the extent that they both received guidelines-based care and they wore the motion sensing equipment. Where they differed was that only the Movement Biofeedback Group had individualised movement pattern/postural rehabilitation, biofeedback and exercises based on the information measured by the motion sensing equipment.

#### Training of participating clinicians

All clinicians participated in a 2-hour technical workshop on how to set up the ViMove system and attach it to a patient. Clinicians in both groups received a laptop loaded with the basic ViMove software, one ViMove sensor unit, and were able to receive additional training in the technical set up and attachment of the ViMove device, if they requested it. In total, this occurred on seven occasions, for four clinicians who were distributed approximately evenly between the Movement Biofeedback and Guidelines-based Care Groups.

The Movement Biofeedback Group clinicians also received the ViMove biofeedback software and an additional 4 hours of training in identifying movement or postural dysfunctions, understanding the software reports, conducting the live training and programming the biofeedback. Two Movement Biofeedback Group clinicians also requested and received some additional training in the technical aspects of live training and programming the biofeedback.

### Outcomes

Outcomes were measured at baseline (Week 0), during the 10-week treatment period (Weeks 1, 3, 6, 8, 10) and during the follow-up period 12 months after baseline (Weeks 12, 26 and 52). All outcomes were measured at every time period, except patient-reported Global Impression of Change, which was measured only at 12 months. Outcomes during the follow-up period were measured via postal questionnaires.

### Primary outcomes

There were three primary outcomes that were measured via patient self-report questionnaires: activity limitation assessed in two ways and pain intensity.

Pain-related activity limitation was measured using both the Roland Morris Disability Questionnaire (RMDQ) and the Patient-Specific Functional Scale (PSFS). The 23-item version [50] of the RMDQ (RMDQ-23) was used to measure condition-specific activity limitation, this version being able to accommodate back-related leg pain. The RMDQ-23 is the most commonly used questionnaire for measuring this construct in people with LBP [51], and has demonstrated a reliability, responsiveness and validity comparable to the available alternative questionnaires [52–54]. Using proportional recalculation, RMDQ-23 scores were transformed into a 0–100 scale (0 = no activity limitation, 100 = maximum activity limitation) [55].

Using the PSFS, patients nominated three functional activities that were important to them and with which they were experiencing some activity limitation (original metric: a 0–10 scale for each item, where 0 = unable to perform activity, 10 = able to perform activity at the same level as before injury or problem). Raw scores were proportionally recalculated and reversed to create a 0–100 scale (0 = no activity limitation, 100 = maximum activity limitation), comparable to the other primary outcome measures. The PSFS has been shown to be valid for group-level change comparisons, between-group discrimination [56] and it is more responsive than the RMDQ-23 for people with low levels of activity limitation [57].

Pain intensity was measured using the average score (0 to 100 scale) of the Quadruple pain Visual Analogue Scale (QVAS), which consisted of four questions (a) ‘What is your back pain intensity right now?’, (b) ‘What was your typical or average pain?’, (c) ‘What was your pain level at its best?’, and (d) ‘What was your pain level at its worst?’. The reference time periods for the last three questions at baseline was ‘over the last 6 months’ and was ‘since your last visit’ at all other assessment time points. The anchors for all four questions were 0 = ‘No pain’ and 100 = ‘Worst possible pain’. Visual analogue scales have been shown to have good reliability [58] and validity for measuring pain intensity [59, 60].

### Secondary outcomes

There were eight secondary outcome measures that were patient-reported on daily diary cards during the treatment period: (i) daily pain score, (ii) LBP analgaesic use, (iii) number of pain-free and medication-free days, (iv) LBP recurrence, (v) time away from work or usual daily activity, (vi) care seeking for LBP outside of the treatment in the trial, (vii) fear of movement, and (viii) patient global impression of change. Change in range of movement over the treatment period was an additional secondary outcome measure, recorded by the ViMove motion-sensor system.

Participating patients completed a diary card at the end of each day that included a number of questions and this diary card was reviewed by their participating clinician at each consultation. One of these questions was a *daily pain score* ‘Considering the day overall, on a scale of 0 to 10, how would you rate your low back pain?’ (0 = no pain, 10 = very severe pain). *LBP analgaesic use* was assessed by two questions on the daily diary card ‘Did you take any pain medication today?’ (yes/no), and ‘How many different pain products did you take today?’ (patients’ wrote the number). *Number of pain and medication free days* was calculated from patients’ responses to the daily pain score and analgaesic use questions on their daily diary card. *Recurrence of LBP* was assessed by the daily diary card question ‘Have you re-injured your back today or had a recurrence of your pain?’ (yes/no). Recurrence was defined as ‘a period of increased pain lasting at least 24 hours’ [61]. *Time away from work or usual daily activity* was self-reported by patients as the number of days off work or of non-participation in their usual social role due to LBP (a health economic outcome). *Care seeking for LBP* was self-reported by patients as the number of health practitioner visits in which they sought care for LBP after the treatment period (also a health economic outcome).

Fear of movement was measured using the Fear Avoidance Beliefs Questionnaire physical activity subscale (FABQpa, 0 to 24 scale) [62]. The FABQpa is a widely used outcome measure, with high internal consistency, construct and predictive validity [63, 64].

Patient Global Impression of Change (PGIC) was measured on a seven-point ordinal Likert scale at the 12-month time-point only (Very much improved, Much improved, Minimally improved, No change, Minimally worse, Much worse, Very much worse). PGIC has shown high reliability [65] and construct validity [66].

Lumbopelvic range of motion (measured in degrees) was recorded in the upright standing and sitting positions for sagittal (flexion and extension) and coronal (lateral flexion) plane movements using the ViMove device. In the Movement Biofeedback Group, this was assessed during treatment sessions, to inform treatment decisions. In all patients, at baseline and each outcome measurement time point, the ViMove device measured lumbopelvic range of motion in activities of daily living, as an outcome measure.

### Sample size

This study was powered for longitudinal analysis, but not for adjusted single-time point comparisons. A sample size calculation indicated that a total sample of 64 participants would have provided 80 % power to detect an effect size of 0.4 given eight site-level clusters, a mean of eight participants per cluster, a two-tailed alpha of 0.05, one baseline assessment, eight follow-up assessments, a correlation between follow-up assessments within participants of 0.5 and an ICC at site level of 0.01. However, this calculation assumed no missing data within participants, no participant withdrawal and equal numbers of participants at each site. Therefore, a 20 % inflation factor was included to accommodate missing data, withdrawals, an imbalance in cluster sizes, resulting in a total sample size requirement of 98 patients.

#### Blinding

During data analysis, the statistician was blind to group allocation by the use of mock codes for group allocation (0,1). Clinicians were not blind to treatment allocation but clinicians in the Guidelines-based Care Group clusters were blind to the information within the ViMove system, and therefore could not modify their treatment based on this movement sensor technology. Patients were blind to treatment allocation and the directionality of the hypothesis being investigated.

### Statistical methods

Data were initially described in a comparison of baseline scores (mean scores, standard deviations) that also tested differences between groups using linear regression (adjusted for clusters). If data were not normally distributed, comparison was made using ordered logit regression, clustered by site.

Next, mixed-effects, multi-level, generalized linear model analysis was performed, adjusted for (fixed effects) baseline scores for the outcome of interest, age, gender, duration of back pain, time since baseline consultation, and (random effects) cluster, clinician and individual patient. For each outcome measure, longitudinal models were created to determine the group effect and the time-by-group interaction effect. Each model was tested to determine if three assumptions about the random errors were met: (i) normal distribution, (ii) constant variance (homoscedasticity), and (iii) zero mean (unbiased). Beta coefficients and Intraclass Correlation Coefficients were reported for each outcome, along with their 95 % confidence intervals (95 % CI) and p-value.

Change in range of motion was calculated using the same multi-level regression analysis, from the standard deviations of the range of motion for the sagittal (flexion and extension) and coronal (lateral flexion) planes recorded during each patient’s wearing of the ViMove device in their activities of daily living. The standard deviation of the range of motion was used to more accurately capture the variation of movements performed during their normal functional activities, under the assumption that participants would show higher variation (and thus higher standard deviation of movements) as their activity limitation improved. To account for movement variability in standing and sitting positions, we computed separate standard deviations for each of these postural positions.

As the Patient Global Impression of Change outcome was only assessed at 12-months, these data were reported descriptively and the (unadjusted for clustering) number needed to treat was calculated using the dichotomised score threshold of those ‘very much improved’ and ‘much improved’ versus all other responses.

The primary outcomes at the 3- and 12-month time points were also reported for both groups (point estimates of the mean, mean improvement from baseline, percentage improvement from baseline, number of patients who improved by >30 % of their baseline score) [67], and also across the groups (difference between group means, difference in percentage improvement, and comparison between groups of probability of improving by >30 % of baseline score (expressed as a risk ratio)). However, as this pilot study was not powered for adjusted comparisons of single time-point outcomes, these results were only reported for descriptive purposes and were not tested for statistical significance. Similarly, the confidence intervals for the crude risk ratios should be interpreted with caution, as they are not adjusted for any baseline imbalances or clustering effects.

All statistical analyses were performed using Stata version 12.1 (Stata Corp, College Station, Texas, USA). Graphs were created using Excel 2011 (Microsoft Corp, Redmond, Washington, USA) or Adobe Indesign CS6 (Adobe Systems, San Jose, California, USA).

## Results

### Participant flow

**Fig. 2:**
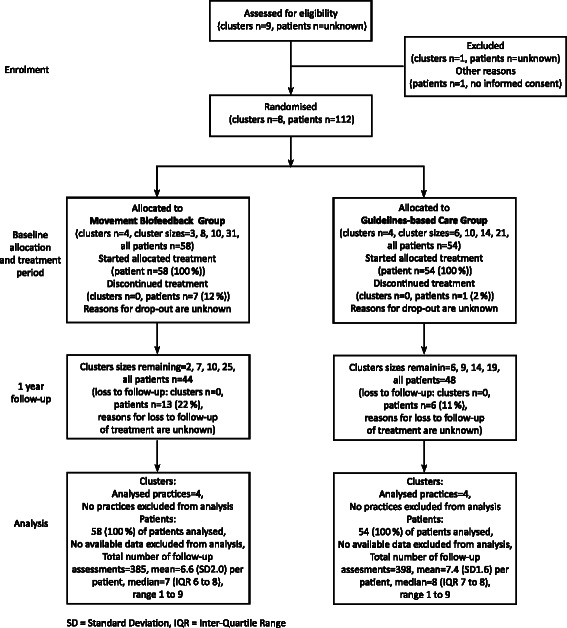
Trial flow diagram

The participant flow chart is shown in Fig. 2. Of the 112 patients recruited, 58 participants (52 %) were enrolled in the Movement Biofeedback Group and 54 participants (48 %) in the Guidelines-based Care Group. Eighty percent had an episode duration greater than 12 weeks, and therefore most patients had eight consultations over the 10-week treatment period. No data were available detailing how many eligible patients were not invited by the recruiting clinicians, the number or characteristics of patients who declined participation, nor the reasons for drop-out or loss to follow-up.

#### Recruitment

Patients were recruited between November 2009 and September 2012, and the follow-up assessments were conducted up until June 2013. Almost all patients provided written informed consent at the initial consultation, and trial-specific treatment commenced at that time, including the initial wearing of the motion-sensor device. For the remaining few patients, these occurred at the second consultation, which became the index consultation for the trial. They wore the ViMove motion-sensor system for 4 hours or more in almost all the measurement sessions of their activities of daily living (Movement Biofeedback Group 94 % of all sessions, Guidelines-based Care Group 93 %). The trial ended when the required sample size had been exceeded and all their 12-month follow-up data had been measured.

#### Baseline data

The baseline demographic and clinical characteristics of both groups are shown in Table 1. The groups differed from each other on age (on average, participants in the Movement Biofeedback Group were 9 years younger than in the Guidelines-based Care Group) and age was adjusted for in all longitudinal analyses.

**Table 1:** Baseline characteristics

	Movement Biofeedback Groupn = 58	Guidelines-based Care Groupn = 54	p-value
Age (years, mean)	39 (SD 12)	48 (SD 12)	0.013
Gender (women, proportion)	30 (52 %)	31 (57 %)	0.729
Pain episode duration (weeks, median)*	52 (IQR 17, 52)	52 (IQR 16, 312)	0.184
Activity limitation (RMDQ-23) (0–100 scale, mean)	51.1 % (SD 38.1)	49.1 % (SD 30.1)	0.758
Activity limitation (PSFS) (0–100 scale, mean)	60.2 (SD 10.1)	57.9 (SD 34.8)	0.660
Pain intensity (QVAS) (0–100 scale, mean)	60.0 (SD 23.6)	61.0 (SD 6.6)	0.758
Fear of movement (FABQpa) (0–24 scale, mean)	13.8 (SD 6.8)	14.4 (SD 8.0)	0.674

RMDQ-23 = Roland Morris Disability Questionnaire (23 item version) where low scores are better

PSFS = Patient Specific Functional Scale, converted to a 0–100 scale where low scores are better

QVAS = Average of four pain intensity VAS scales, where low scores are better

FABQpa = Fear Avoidance Beliefs Questionnaire (physical activity subscale)

*Data presented are median (IQR) due to skew in data, group comparison undertaken using ordered logit regression clustered by site

#### Co-interventions administered

The co-interventions received in addition to advice on staying active and general self-management of back pain (both groups), and the technology-assisted movement/postural re-education received by the Movement Biofeedback Group, are summarised in Table 2.

**Table 2:** Co-interventions received during treatment period

	Movement Biofeedback Group		Guidelines-based Care Group	
Intervention Type	Number of patients receiving each intervention type	Mean number of treatments per patient	Number of patients receiving each intervention type	Mean number of treatments per patient
Advice or education	18 (31.0 %)	0.58 (SD 1.02)	19 (35.2 %)	1.48 (SD 2.62)
Exercise	32 (55.2 %)	1.40 (SD 1.77)	40 (74.1 %)	4.78 (SD 3.25)
Imaging	3 (5.2 %)	0.07 (SD 0.32)	8 (14.8 %)	0.13 (SD 0.34)
Manual Therapy	36 (62.1 %)	1.89 (SD 1.98)	30 (55.6 %)	1.26 (SD1.73)
Medication	6 (10.3 %)	0.16 (SD 0.53)	36 (66.7 %)	2.91 (SD2.96)
Other	15 (25.9 %)	0.35 (SD 0.74)	8 (14.8 %)	0.20 (SD0.56)
Taping or Bracing	1 (1.7 %)	0.02 (SD 0.13)	2 (3.7 %)	0.02 (SD 0.14)

#### Numbers analysed

To adhere to the intention-to-treat principle, all recruited patients contributed all their measured data regardless of drop out (longitudinal analysis manages missing data well), and analysis was by original assigned group. As patients could have had six to eight treatment sessions, and as treatments could have varied on which week they occurred, these data are somewhat statistically unbalanced. This did not affect the statistical integrity of the longitudinal analyses, but did create some arbitrariness regarding the allocation of data in the visual figures that diagrammatically represent the clinical course of the two groups, as individual patient consultations were simply allocated to the closest descriptive week.

### Outcomes and estimation

#### Primary outcomes

**Fig. 3:**
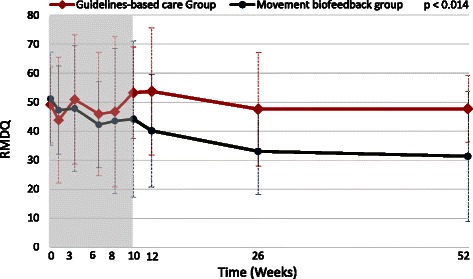
Mean outcomes for activity limitation (Roland Morris Disability Questionnaire scores)

**Fig. 4:**
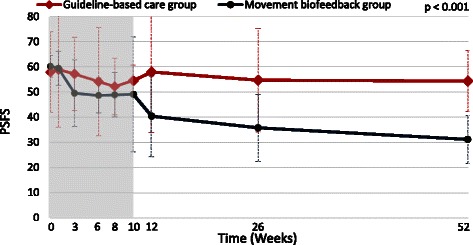
Mean outcomes for activity limitation (Patient-Specific Functional Scale scores)

**Fig. 5:**
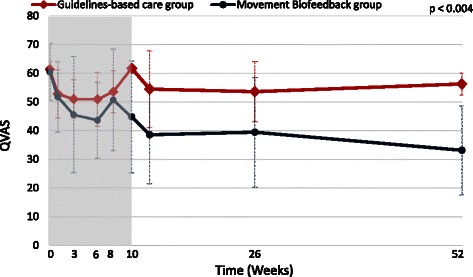
Mean outcomes for pain intensity (Quadruple Visual Analogue Scale scores)

The estimated effects of the Movement Biofeedback intervention are shown numerically in Table 3 for the primary outcome measures and in Table 4 for the secondary outcome measures. Results for the primary outcome measures at all time-points are also summarised visually in Figs. 3, 4 and 5. All of the primary outcomes were similar at baseline but over time favoured the clinical course of the Movement Biofeedback Group at a statistically significant level. Of note is that the differences present at the end of the treatment period persisted over the follow-up period, and at the 12-month period, appeared to have continued to grow.

**Table 3:** Results for primary outcome measures

	Activity limitation (RMDQ23: 0 to 100 scale)	Activity limitation (PSFS: 0 to 100 scale)	Pain intensity (QVAS: 0 to 100 scale)
Preplanned analysis - Clinical course*			
*Movement Biofeedback Group effect* ^*#*^			
Beta coefficient (95 % CI)	−7.1(−12.6 to −1.6) p < 0.014	−10.3(−16.6 to −3.9) p = 0.001	−7.7(−13.0 to −2.4) p < 0.004
*Group effect Intraclass Correlation Coefficients*			
Clinics	0.00	0.04	0.00
Clinicians	0.00	0.00	0.00
Patients	0.50	0.37	0.55
*Movement Biofeedback Group-by-time (per 100 days) effect* ^*#*^			
Beta coefficient (95 % CI)	−3.5(−5.2 to −2.2) p < 0.001	−4.7(−7.0 to −2.5) p < 0.001	−4.8(−6.1 to −3.5) p < 0.001
*Group-by-time effect Intraclass Correlation Coefficients*			
Clinics	0.00	0.00	0.00
Clinicians	0.00	0.00	0.00
Patients	0.51	0.38	0.59
Analysis n=	Sites = 8	Sites = 8	Sites = 8
	Clinicians = 8	Clinicians = 8	Clinicians = 8
	Participants = 106	Participants = 96	Participants = 106
	Assessments = 644	Assessments = 524	Assessments = 650
Additional analysis - Unadjusted comparison at individual time points**			
*3-month outcomes*			
*Movement Biofeedback Group*			
Mean (95 % CI)	40.1 (20.7 to 59.5)	40.0 (24.0 to 56.0)	39.5 (21.4 to 55.7)
Mean improvement from baseline (95 % CI)	11.4 (7.3 to 15.5)	18.9 (6.1 to 31.7)	22.1 (13.6 to 30.5)
n (%) of patients who improved by ≥30 % of baseline score	15 (43 %)	16 (55 %)	17 (49 %)
Analysis n=	35	29	35
*Guidelines-based Care Group*			
Mean (95 % CI)	53.7 (31.8 to 75.6)	58.0 (34.0 to 82.0)	54.5 (41.1 to 67.8)
Mean improvement from baseline (95 % CI)	−1.6 (−8.4, 5.2)	1.3 (−8.7, 11.4)	9-4 (2.4 to 16.3)
n (%) of patients who improved by ≥30 % of baseline score	6 (16 %)	12 (40 %)	12 (32 %)
Analysis n=	37	30	37
*Difference between group means* ^*#*^	−13.0 (−18.5 to 7.5)	−17.6 (−28.9 to −6.3)	−12.7 (−20.2 to −5.1)
*Comparison between groups of probability of improving by ≥30 % = risk ratio (95 % CI)* ^†^	2.6 (1.2 to 6.0)	1.4 (0.8 to 2.4)	1.5 (0.8 to 2.7)
*12 month outcomes*			
*Movement Biofeedback Group*			
Mean (95 % CI)	31.3 (8.9 to 53.7)	31.0 (22.0 to 41.0)	33.1 (17.7 to 48.6)
Mean improvement from baseline (95 % CI)	19.7 (15.4 to 24.0)	28.1 (20.4 to 35.9)	27.5 (21.7 to 33.3)
n (%) of patients who improved baseline score by ≥30 % of baseline score	26 (60 %)	31 (78 %)	30 (68 %)
Analysis n=	43	40	44
*Guidelines-based Care Group*			
Mean (95 % CI)	47.7 (36.2 to 59.2)	54.0 (42.0 to 64.0)	56.2 (52.4 to 60.1)
Mean improvement from baseline (95 % CI)	1.5 (−4.2 to 7.2)	3.2 (−8.6 to 15.0)	5.4 (3.3 to 7.4)
n (%) of patients who improved baseline score by ≥30 % of baseline score	12 (25 %)	12 (32 %)	10 (21 %)
Analysis n=	47	38	48
*Difference between group means* ^*##*^	−18.2 (−23.1 to −13.2)	−24.9 (−34.7 to −15.2)	−22.2 (−26.4 to −17.9)
*Comparison between groups of probability of improving by ≥30 % = risk ratio (95 % CI)* ^†^	2.4 (1.4 to 4.1)	2.5 (1.5 to 4.0)	3.3 (1.8 to 5.9)

*Calculated by use of multilevel mixed-effects linear regression adjusted for baseline value of the outcome measure, age, gender, and duration of back pain episode (fixed effects) and treatment site, clinician and individual patient (random effects)

**This pilot cluster trial was not powered for individual time point comparisons and therefore these unadjusted descriptive results were not tested for statistical difference

^*#*^The *main effect of group* indicates the average difference between the groups across treatment and outcome time points. The *time-by-group interaction effect* indicates the average difference between the groups in the rate of change over time

^*##*^Difference between group means = Guidelines-based Care Group minus Movement Biofeedback Group. Analyses adjusted for clustering by site and robust 95 % confidence intervals used

^†^Crude risk ratio = Movement Biofeedback Group / Guidelines-based Care Group. These unadjusted confidence intervals should be cautiously interpreted, as they do not account for any baseline imbalances or clustering effects

RMDQ-23 = Roland Morris Disability Questionnaire (23 item version) where low scores are better, PSFS = Patient Specific Functional Scale converted to a 0–100 scale where low scores are better, QVAS = Average of four pain intensity VAS scales where low scores are better

**Table 4:** Results for secondary outcome measures

	Analysis n=	*Movement Biofeedback*	*Movement Biofeedback*	*End of treatment period*
		*Group effect* ^#^	*Group by time effect* ^#^	*Mean (SD)*
		Beta coefficient (95 % CI)		
			Beta coefficient *(per 10 days in the treatment period)* (95 % CI)	
Daily pain score	Sites = 8	−0.62 (−1.25 to 0.01)	−0.051 (−0.075 to −0.026)	*Movement Biofeedback Group*
*(0 to 10 scale)*	Clinicians = 8			
	Participants = 98	p = 0.053	p < 0.001	
	Assessments = 6,036			*4.26 (3.44 to 4.99)*
				*Guidelines-based Care Group4.54 (3.88 to 5.19)*
LBP recurrence (difference in proportions of days with reported recurrence)	Sites = 8	−0.018 (−0.129 to 0.093)	0.003 (−0.002 to 0.008)	*Movement Biofeedback Group*
	Clinicians = 8			
	Participants = 100	p = 0.752	p = 0.263	
	Assessments = 5,999			0.230 (0.098 to 0.362)
				*Guidelines-based Care Group*
				*0.173 (0.070 to 0.276)*
Analgesic use	Sites = 8	0.056 (−0.099 to 0.211)	−0.007 (−0.013 to −0.002)	*Movement Biofeedback Group*
(difference in proportion of days with reported taking of analgesics)	Clinicians = 8			
	Participants = 98	p = 0.483	p = 0.008	*0.288 (0.137 to 0.440)*
	Assessments = 5,815			
				*Guidelines-based Care Group*
				*0.360 (0.109 to 0.612)*
Number of pain and	Sites = 8	0.054 (0.003 to 0.107)	0.004 (0.002 to 0.007)	*Movement Biofeedback Group*
medication free days	Clinicians = 8			*0.064 (−0.034 to 0.163)*
				*Guidelines-based Care Group*
				*0.036 (−0.006 to 0.077)*

^#^The *main effect of group* indicates the average difference between the groups across treatment and outcome time points. The *time-by-group interaction effect* indicates the average difference between the groups in the rate of change over time. Both calculated by use of multilevel mixed-effects linear regression adjusted for baseline value of the outcome measure, age, gender, and duration of back pain episode (fixed effects) and treatment site, clinician and individual patient (random effects)

FABQpa = Fear Avoidance Beliefs Questionnaire (physical activity subscale) where low scores are better

Statistically significant p-values are bolded

The *main effect of group*, indicating the average difference between the groups across treatment and outcome time points, significantly favoured the Movement Biofeedback Group on all the primary outcomes, being 7.1 RMDQ points, 10.3 PSFS points and 7.7 QVAS points in size (confidence intervals reported in Table 3). The *time-by-group interaction effect*, indicating the average difference between the groups in the rate of change over time, also significantly favoured the Movement Biofeedback Group on all the primary outcomes, being 3.5 RMDQ points, 4.7 PSFS points and 4.8 QVAS points in size, for every 100 days since the baseline consultation. In addition, the unadjusted risk ratios for the proportion of patients who improved by a clinically important amount (>30 % of baseline scores) [67], all significantly favoured the Movement Biofeedback Group and ranged from 1.4 to 2.6 at 3 months and from 2.4 to 3.3 at 12-months.

Intraclass Correlation Coefficients were also reported in Tables 2 for each primary outcome, which provide an estimate of the lack of statistical independence between individuals in the same cluster.

#### Secondary outcomes

In the mixed-effects, multi-level, generalized linear model analysis, there were statistically significant group effects on two of the eight secondary outcome measures and significant group-by-time effects on three of these secondary outcomes, all favouring the Movement Biofeedback Group. There were no significant group or group-by-time effects for the following five secondary outcome measures: LBP recurrence, fear of movement, time away from work or usual daily activity, care seeking for LBP, and range of movement.

There were significant group and group-by-time effects on the *daily pain score*. The pain reduction, averaged over the 72-day treatment period, was 0.62 points more for the Movement Biofeedback Group than for the Guidelines-based Care Group. Similarly, for every 10 days in the treatment period, the daily pain score reduced by 0.051 more in the Movement Biofeedback Group than in the Guidelines-based Care Group.

For *LBP analgaesic use*, there was a significant group-by-time effect. For every 10 days in the 72-day treatment period, the proportion of days reported taking analgaesics reduced by 0.007 more in the Movement Biofeedback Group, than in the Guidelines-based Care Group.

There were significant group and group-by-time effects on the *number of pain and medication free days.* The proportion of pain and analgaesic medication free days over the 72-day treatment period was 0.042 more in the Movement Biofeedback Group than in the Guidelines-based Care Group. Also, for every 10 days in the treatment period, the proportion of days reported as not having pain or taking any analgaesics increased by 0.004 more in the Movement Biofeedback Group than in the Guidelines-based Care Group.

Global impression of change was analysed separately using the unadjusted number needed to treat. A larger proportion of participants in the Movement Biofeedback Group reported that they were very much or much improved than in the Guidelines-based Care Group (number needed to treat = 2.8 (95 % CI: 1.9 to 5.8)).

#### Harms

Across the 629 total consultations in which the ViMove devices were worn by the patients, there were 17 instances (2.7 %) of device-related side effects. All involved some form of transient skin irritation from the hypo-allergenic tape used to mount a motion-sensor. These occurred in six Movement Biofeedback Group patients and 11 Guidelines-based Care Group patients but did not preclude wearing the device at the next scheduled outcome measurement time-point.

## Discussion

This cluster-randomised pilot trial investigated whether changing patterns of lumbo-pelvic movement and/or posture using motion-sensor biofeedback in people with LBP would lead to reduced pain and activity limitation, when compared with guidelines-based medical or physiotherapy care. It aimed to: (i) estimate the effect size and its variability, (ii) test the study protocol and procedures, and (iii) provide data (ICCs) to calculate sample size requirements that would allow adjusted individual time-point comparisons in a fully powered cluster-randomised clinical trial. All three aims were achieved; there were significant treatment effects favouring the intervention group on all primary outcomes, insights were gained about refining a protocol for a fully powered trial and ICCs were calculated.

### Treatment effect

Patients in the Movement Biofeedback Group showed significant improvements in the primary outcome measures of activity limitation and pain intensity, compared with those in the Guidelines-based Care Group, as seen by the group effects and group-by-time interaction effects all favouring the Movement Biofeedback Group. The *group effect* indicates the average difference between the groups across treatment and outcome time points, and the *time-by-group interaction effect* indicates the average difference between the groups in the rate of change over time. Furthermore, across all these outcome measures, the additional (unadjusted) percentage improvement in the Movement Biofeedback Group ranged from 15 % to 27 % at 3 months and 35 % to 47 % at 12 months, which were all above the threshold for clinically important difference (>30 % of baseline scores) [67]. Similarly, the unadjusted risk ratios all significantly favoured the Movement Biofeedback Group, indicating that the probability of the Movement Biofeedback Group patients improving by a clinically important amount at 3 months was from 1.4 to 2.6 times more likely than the Guidelines-based Care Group patients, and from 2.4 to 3.3 times more likely at 12 months. These results are unusual and encouraging because they show moderate to large effects at the end of the 10-week treatment period that remained or increased at the 12 month follow-up, in a health condition where interventions typically show small to moderate effects [8] that are not sustained 12 months later [9–11]. Our results suggest that where a relationship between movement and pain can be identified, movement retraining using biofeedback is capable of resulting in sustained improvements in pain and activity limitation, even after treatment finishes, and indicate that a fully powered trial is warranted.

In addition, there was no difference in fear of movement over time between the treatment groups. This is reassuring, as it indicates that the focus in the Movement Biofeedback Group on retraining movement patterns/posture and having six to eight sessions of biofeedback did not increase participants’ fear of movement.

The only other clinical trial of similar individualised movement rehabilitation for persistent LBP, of which we are aware, was recently published by Vibe Fersum et al. [17]. It included movement and postural re-education as part of a comprehensive biopsychosocial approach (Cognitive Functional Therapy). Although it did not use technology to assist in the assessment and management of LBP, and included physiotherapists but not GPs as clinicians, it similarly showed moderate to large effect sizes that persisted over the follow-up period. This similarity of promising results in these two studies in primary care suggests that individualised movement rehabilitation should be further studied, as many aspects of these results remain unaddressed. For example, it is unclear from our study what relative contributions to the results came from the movement rehabilitation and those that came from the use of motion-sensor technology. Theoretically, this technology may provide greater precision of assessment, more specificity in movement re-education, and enhanced de-habituation of dysfunctional movement via biofeedback in daily functional activities. However, this needs to be investigated. It is also not clear what might mediate that treatment effect, such as cognitive, motivational or movement awareness aspects resulting from wearing the motion-sensors. In addition, evidence of a relationship between modifiable movement aberrations and reductions in pain and activity limitation would still leave unaddressed questions about whether movement aberrations precede the onset of pain and involve some causative mechanisms, or whether they are secondary to the onset of pain, or both.

Clinicians participating in the trial conducted by Vibe Fersum et al. [17] had an average of more than 100 hours training in Cognitive Functional Therapy. In our pilot trial, clinicians in the Movement Biofeedback Group had an average of approximately 6 hours training in the use of the device. While the amount of previous exposure to principles of movement rehabilitation in the clinicians in both trials is unknown, it is possible that the precision of patient-specific kinematic information available to clinicians using motion-sensor technology allows some degree of ‘learning by doing’ and this aspect should be investigated.

In previous studies, the effectiveness of movement interventions for LBP, such as exercise, has been modest. It has also not been consistently associated with any particular form of movement intervention [68–75], regardless of whether it involves whole body movements such as aerobic exercise, Pilates, and yoga, or targets the activity of specific muscles such as Transversus Abdominus [76, 26]. One possible explanation for this is that generic ‘one size fits all’ approaches poorly target any movement aberrations that may be present at an individual patient level. However, highly individualised exercise programs that aim to alter lumbo-pelvic kinematics or postural patterns, such as those based on the Alexander Technique [77, 78], the Feldenkrais Method [77] or Pilates [79], have also shown modest and inconsistent effects. One explanation could be that these approaches are too narrow to adequately cover the range of movement dysfunctions seen in a LBP care-seeking population. Another possibility is that some movement dysfunctions are too subtle to be routinely detected outside of laboratory settings by non-expert clinicians, unless assisted by technology such as motion-sensors. A further possibility is that changing movement patterns in people’s habituated daily activities requires measurement and biofeedback during those activities, especially since there is evidence that practice with feedback distributed across time is more effective for learning than concentrated feedback at one time point [80].

Of note is that there were no statistically significant differences between the groups in the variability in range of movement displayed by participating patients during their normal functional activities. This may reflect the finding in the trial conducted by Vibe Fersum et al. [17], that there were no differences between their groups in total range of movement, despite large differences in the primary outcomes. However, the experience of this pilot study taught us that the movement parameters that were classified by clinicians as requiring modification were diverse and individualised, with some people already moving excessively and requiring some aspect of their range of motion to be restricted. Therefore, metrics based on total range of variability in movement may not capture improvements in movement patterns that are associated with less pain during activity. There may be better ways to capture the kinematic characteristics that are important at an individual patient level and determine whether these were improved more in one group than another. One method to do so would be to capture data on which movement characteristics each clinician judged should be targeted in each patient (by monitoring what movements were programed for biofeedback). This would allow the motion-sensor technology to be used to measure the extent to which those movement re-education goals were achieved. We did not collect the data to analyse this in our pilot study but it would be ideal to collect these in a fully powered trial.

### Study protocol and procedures

The study protocol and a number of the procedures evolved during this pilot trial. These included a refinement of the treatment approach and software, as well as greater clarity about how to measure appropriate outcomes. As this pilot study investigated the application of new technology, it was inevitable that the experience would teach us better ways of presenting information to clinicians and better ways of clinicians using that information. Testing those ways within this pilot study was a component of preparing for a fully powered study.

Based on the experience and results of this study, there are a number of features that would be ideal to incorporate in the protocol of a fully powered trial. For example, it would be pragmatic to add to the inclusion criteria the need for all participants to display some form of movement-related pain [17] (pain aggravated or relieved by movement). That is because the intervention is designed to target movement-related pain and patients without this characteristic are likely to dilute the treatment effect size. It would also be sensible to use the more detailed treatment protocol and software that evolved throughout this study, as these show face validity for providing greater specificity for targeting abnormal movement/posture. In addition, adjusted statistical comparisons of multiple individual time-point outcomes would provide greater certainty about the results and allow more direct comparisons with other trials.

It would also be useful to collect data identifying each patient’s progress towards attaining the ‘more optimal movements/postures’ that were targeted in their particular case, as this would allow examination of a ‘dose-response’ relationship between improvement in movement and improvement in pain and activity limitation. Perhaps this could be formalised by the creation of a ‘Patient-Specific Movement Scale’.

It would also be ideal if recruitment were not performed by the treating clinicians, as in the context of a cluster-randomised controlled trial, this can introduce the potential for selection bias. One way that this could be done would be for potential participants to answer recruitment advertisements and then be randomised to clusters, however this would introduce the artificiality of participants not having sought care from the clinician of their choice. In addition, it would be helpful to measure whether patients guessed which group they were allocated to, as an estimate of patient unblinding.

### Data to calculate sample size requirements

The statistical power of a clustered sample, in which participants are randomised at a group level but analysed at an individual level, is a function of (i) the relatedness of clustered data (Intraclass Correlation Coefficient), (ii) the sample size of the clusters, and (iii) the total sample size (the number of patients per cluster times the number of clusters) [40]. The Intraclass Correlation Coefficients in the results of this trial greatly facilitate precision in sample size requirement calculations for subsequent trials in similar settings.

For example, we could now plan for a cluster-randomised trial to detect a 0.5 standardised effect size for the Roland Morris Disability Index primary outcome at a single time point. If we used only a single 12-month follow-up measure, had 12 clinics/clusters, employed a two-tailed alpha of 0.05, we could conservatively use a 0.01 ICC value for the clinic level ICC value based on our pilot study finding of 0.00 (Table 3) to estimate that each cluster would need to have 12 month data from approximately 11.5 participants to have 80 % power. Knowing that we experienced approximately 20 % attrition between recruitment and 12-month follow-up in our pilot study would mean we would aim to recruit 14 participants per cluster, with a total sample size requirement of 168 patients.

### Limitations

The study had a number of limitations. This pilot trial involved co-funding and participation by the device manufacturer. This was necessary to secure the external funding that made this study possible and was very useful in the training/supporting of clinicians with this new technology and in the refining of the software/treatment protocol. This industry involvement can raise concerns that the study objectivity might have been compromised. However, it was the role of the authors to ensure that all analysis was performed per protocol and not by a company representative, and that the interpretation of the findings was completely independent of both the industry and governmental sponsors. Once the trial protocol was approved by the ethics committees, there were no changes to the primary outcome measures or statistical analysis protocol. Neither sponsor was sent, or requested, any version of this paper prior to publication. In addition we, and the involved ethics committees, the Trial Steering and Data Monitoring Committee and the independent external auditor, all provided governance functions designed to safeguard that the trial maintained its scientific rigour. The independent external audit included verification that every measurement of every patient recruited into the trial was analysed and contributed to the results.

Over the 12-month follow-up period, the Guidelines-based Care Group improved minimally (RMDQ-23 3.1 %, PSFS 5.5 %, QVAS 8.9 %), whereas it is typical for similar LBP control groups to improve by 10 % or more, regardless of treatment [17]. One explanation for this may be that the lack of clinician blinding resulted in Guidelines-based Care Group clinicians not delivering their intervention with the same enthusiasm as clinicians in the Movement Biofeedback Group (performance bias). It is also possible that there was some selection bias, although the only significant difference measured between the groups was on age and as the longitudinal analyses were adjusted for this baseline imbalance, it may not have affected the estimates of effect. We cannot know whether either a performance or selection bias was present or not.

In addition, there was a difference in the reference time period for QVAS at baseline compared with the reference period used at the follow-up time-points, which potentially may have biased the results. However as the size of the QVAS effect was very similar to that for the RMDQ-23, any impact was likely to have been minimal.

We had intended to measure range of motion in the horizontal plane (rotation) but technical limitations of the ViMove motion-sensors resulted in this being impractical. As rotation has a smaller range of movement in the lumbar spine than movement in the other two planes, and as no measured movements in those planes was significantly different between the groups, this limitation was likely to have been of no practical consequence.

The generalisability of these results is enhanced by the trial’s cluster randomised design, as this directly adjusts for the influences of clinician and site, and by the inclusion of eight sites, albeit that all sites were within one metropolitan area. However, the applicability of the results outside of the research context is constrained by the need for clinicians to be trained in the use of the ViMove system and have access to it, and to be familiar with movement re-education approaches.

## Conclusions

This cluster-randomised pilot clinical trial found evidence that changing patterns of lumbo-pelvic movement and/or posture using motion-sensor biofeedback in people with low back pain leads to reduced pain and activity limitation, when compared with guidelines-based medical or physiotherapy care and placebo. These treatment effects were moderate to large at the end of the 10-week treatment period and were sustained or increased at the 12-month follow-up. Retraining movement patterns/posture using movement biofeedback did not increase participants’ fear of movement. The study protocol and procedures also evolved during this pilot trial, including the treatment approach and software used with wearable motion-sensors. These insights will allow greater precision of treatment targeting in a fully powered trial and the measurement of additional appropriate outcomes. The results provided useful data to calculate sample size requirements that would allow adjusted individual time-point comparisons in a fully powered cluster randomised clinical trial. Collectively, these results indicate that motion-sensor biofeedback may have a role in treating people with back pain and thus, a fully powered trial is warranted.

### Availability of Supporting Data

Supporting data is available on request from the first author.

## Abbreviations

FABQpa: Fear Avoidance Beliefs Questionnaire physical activity subscale
LBP: Low back pain
PSFS: Patient-Specific Functional Scale
PGIC: Patient Global Impression of Change
QVAS: Quadruple pain Visual Analogue Scale
RMDQ-23: 23-item version of the Roland Morris Disability Questionnaire
